# Sarcomas of the extremities and the pelvis: comparing local recurrence after incisional and after core-needle biopsy

**DOI:** 10.1186/s12957-021-02481-2

**Published:** 2022-01-11

**Authors:** Alexander Klein, Christof Birkenmaier, Julian Fromm, Thomas Knösel, Dorit Di Gioia, Hans Roland Dürr

**Affiliations:** 1 Department of Orthopaedics and Trauma Surgery, Musculoskeletal University Center Munich (MUM), University Hospital, LMU Munich, 81377 Munich, Germany; 2 Department of Orthopaedics, Physical Medicine and Rehabilitation, University Hospital, LMU Munich, Marchioninistr. 15, 81377 Munich, Germany; 3 Institute of Pathology, University Hospital, LMU Munich, 81377 Munich, Germany; 4 Department of Medicine III, University Hospital, LMU Munich, 81377 Munich, Germany

**Keywords:** Sarcoma, Incisional biopsy, Core needle biopsy, Local recurrence, Bone, Soft tissue

## Abstract

**Background:**

The degree of contamination of healthy tissue with tumor cells during a biopsy in bone or soft tissue sarcomas is clearly dependant on the type of biopsy. Some studies have confirmed a clinically relevant contamination of the biopsy tract after incisional biopsies, as opposed to core-needle biopsies. The aim of our prospective study was to evaluate the risk of local recurrence depending on the biopsy type in extremity and pelvis sarcomas.

**Methods:**

We included 162 patients with a minimum follow-up of 6 months after wide resection of extremity sarcomas. All diagnostic and therapeutic procedures were performed at a single, dedicated sarcoma center. The excision of the biopsy tract after an incisional biopsy was performed as a standard with all tumor resections. All patients received their follow-up after the conclusion of therapy at our center by means of regional MRI studies and, at a minimum, CT of the thorax to rule out pulmonary metastatic disease. The aim of the study was the evaluation of the influence of the biopsy type and of several other clinical factors on the rate of local recurrence and on the time of local recurrence-free survival.

**Results:**

One hundred sixty-two patients with bone or soft tissue tumors of the extremities and the pelvis underwent either an incisional or a core-needle biopsy of their tumor, with 70 sarcomas (43.2%) being located in the bone. 84.6% of all biopsies were performed as core-needle biopsies. The median follow-up time was 55.6 months, and 22 patients (13.6%) developed a local recurrence after a median time of 22.4 months. There were no significant differences between incisional and core-needle biopsy regarding the risk of local recurrence in our subgroup analysis with differentiation by kind of tissue, grading of the sarcoma, and perioperative multimodal therapy.

**Conclusions:**

In a large and homogenous cohort of extremity and pelvic sarcomas, we did not find significant differences between the groups of incisional and core-needle biopsy regarding the risk of local recurrence. The excision of the biopsy tract after incisional biopsy in the context of the definitive tumor resection seems to be the decisive factor for this result.

## Background

The scientific discussion regarding the standards for diagnostic biopsies in sarcomas of the extremities is ongoing. The choice between core-needle biopsy (CNB) and incisional biopsy (IB) depends not only on medical indications (e.g., location of the tumor, proximity of the nerve and vessel routs), but also on the experience of the surgeon and the pathologist and on the infrastructure. The diagnostic accuracy appears to be equal with both types of biopsy and for both, soft tissue and bone sarcomas [1–3]. Other studies have shown that no relevant tumor cell seeding occurs in the biopsy tract during CNB and that not excising the biopsy tract does not increase the risk of local recurrence (LR) [4, 5]. As a consequence, if excising the biopsy tract is not regarded as mandatory in CNB, less patients have to undergo plastic surgery for closure of resulting larger defects. Tissue sampling can also be done much faster and without general anesthesia, if CNB is used.

Previous studies have investigated the contamination of the biopsy tracts in soft tissue and bone sarcomas with both kinds of biopsies. The significantly higher tumor contamination of the biopsy tracts in IB cases suggests a significantly higher risk of LR [6]. While an excision of the tract in CNB cases does not seem to be necessary with respect to local recurrence-free survival (LRFS), the wide excision of IB tracts is important. There are not many studies investigating the influence of biopsy type on local recurrence with a relevant number of included cases [7–9]. Other factors influencing LRFS or overall survival (OS), like margins, grading, and perioperative multidisciplinary approach, are well known [10].

We prospectively recorded the follow-up of surgically treated patients at our institution, who had a biopsy between 2013 and 2018. The subject of this study was two questions: Does the type of biopsy influence the risk of LR and LRFS? Are there differences between subgroups (bone/soft tissue sarcoma, perioperative therapy) at our cohort?

## Methods

Patients, treated between 2013 and 2018 at our department, were evaluated. We included 174 patients in this study. Inclusion criteria were:

- Final diagnosis of a primary sarcoma of bone or soft tissue of the extremities and pelvis
- Biopsy performed at our institution
- Final surgery performed at our institution
- Wide (R0) resection of the tumor
- Follow-up > 6 months

A team of two experienced surgeons performed all biopsies and resections of the tumors. Two different biopsy approaches were used: CNB and IB. A CNB without guidance was performed in cases of well-palpable tumors without risk of injury of vessels or nerves. Computed tomography (CT) or sonographic guidance in CNB was used for deep tumors. IB was used in cases with thick cortical bone that required drilling or with anatomically complex location of soft tissue sarcomas. After confirmation of the diagnosis, staging investigations were performed by means of magnetic resonance imaging (MRI) of the complete affected compartment and CT of the thorax and abdomen. In cases of Ewing sarcomas or osteosarcomas, positron-emitting tomography-CT (PET/CT) was used for staging. Depending on the entity, resection margins, and additional factors, neo-/adjuvant local radiotherapy or chemo-/targeted therapy was individually decided in the setting of an interdisciplinary tumor board.

Bone sarcomas were treated according to the established standards: chondrosarcomas underwent primary surgical resection (only dedifferentiated chondrosarcomas underwent an interdisciplinary approach). High-grade osteosarcomas were treated according to the EURO-B.O.S.S [11]. protocol or EURAMOS-1 protocol [12]; Ewing sarcomas underwent an interdisciplinary therapy according to the Euro-EWING 99 trial [13].

High-grade soft tissue sarcomas were treated interdisciplinary according to the guidelines of the European Society for Medical Oncology (ESMO) [14] with the additional option of regional hyperthermia [15]. Neo- or adjuvant radiotherapy was added in dependence of the entity, location, size, grading, and resection margins.

Two sarcoma-experienced pathologists performed all histopathological studies. Every sarcoma biopsy sample and specimen was characterized according to the Fédération Nationale des Centres de Lutte Contre le Cancer (FNCLCC) grading system: G1 as low-grade sarcoma and G2 and G3 as high-grade sarcoma. Tumor-free resection margins were defined as R0 resection, contaminated margins as R1, and intralesional margins as R2 resections. Only patients with R0 resections were included in this study (see the inclusion criteria).

In cases of CNB, we did not excise the biopsy tract during the tumor resection. In IB, we included the biopsy tract into a wide resection with a safety distance of ≥ 2 cm in all directions.

For follow-up, every sarcoma patient underwent regional MRI investigations (in some cases, e.g., myxoid liposarcoma additional extended MRI of the spine and pelvis) and CT of the thorax (in some cases also CT of the abdomen) in regular intervals: for the first year, every 3 months postoperatively, then either every 3 or 6 months depending on the type of sarcoma.

To examine our results for statistical significance, analysis was performed using the chi-square test set at a 95% confidence interval. For groups smaller than 20 individuals, we used Fisher’s exact test. Multivariate analysis (Cox proportional hazards regression) was used for the evaluation of a potential influence of patient characteristics on the risk of LR and on LRFS. The level of significance was set at less than 0.05. Local recurrence-free survival was calculated according to the Kaplan-Meier method, using the log-rank test for significance testing. The data analysis software used was IBM® SPSS® Statistics 25.

This study was approved by the institutional ethics committee. Written consent was obtained from all surviving patients included in this study. For non-surviving patients, data were irreversibly anonymized as recommended by the ethics committee.

## Results

### Total cohort

**Fig. 1:**
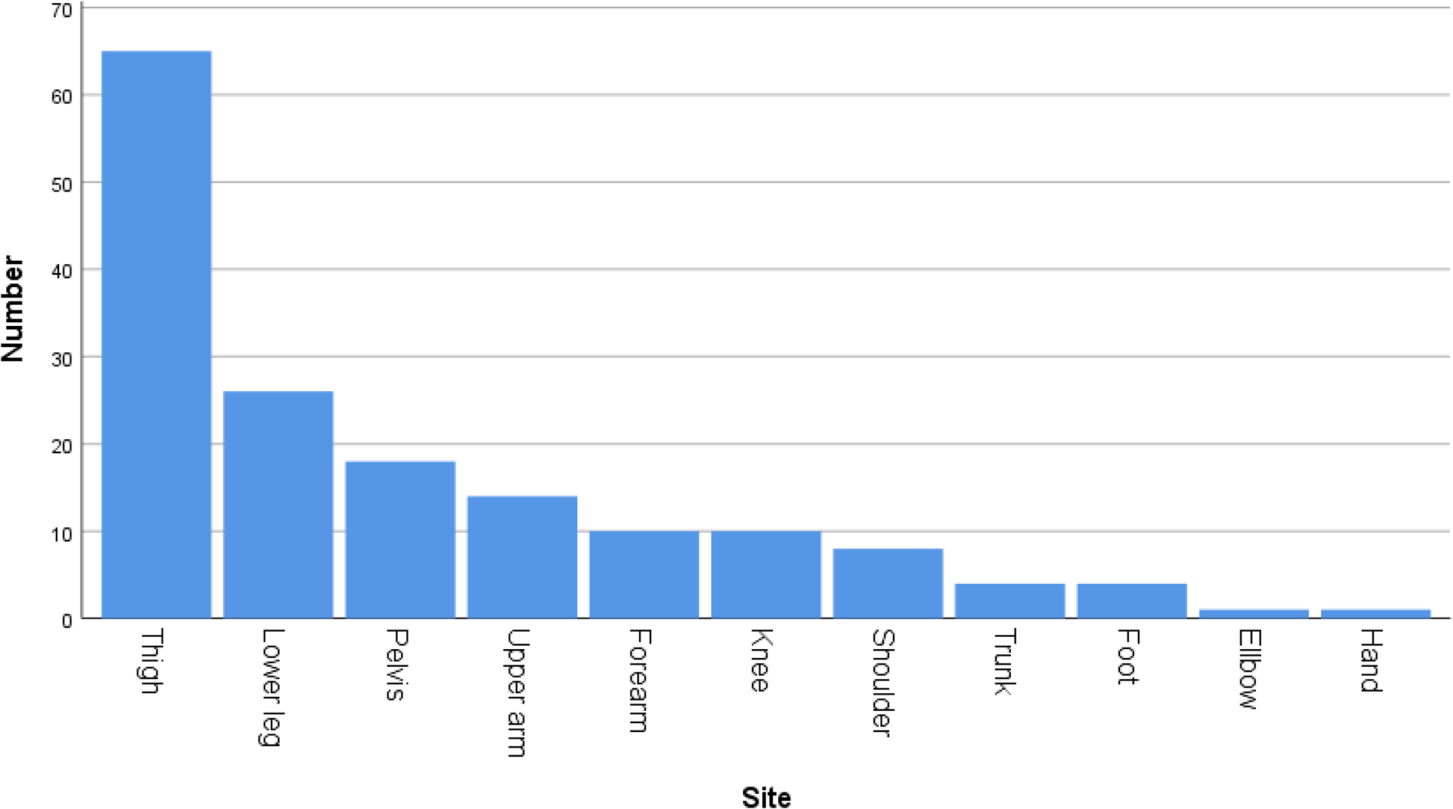
Location of the included tumors (*n* = 162)

**Fig. 2:**
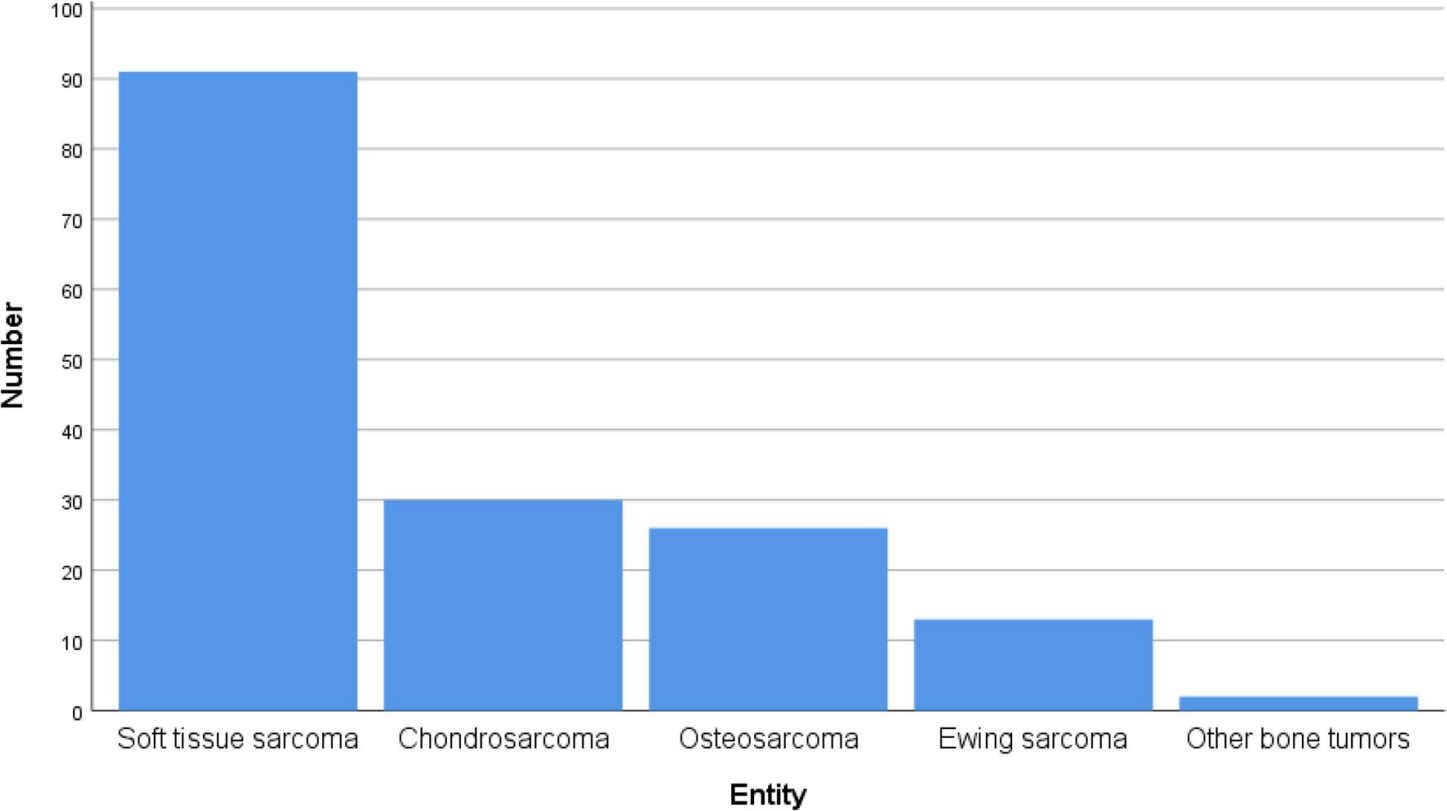
Entities in the patient cohort (*n* = 162)

We included 174 patients, who met our inclusion not our exclusion criteria. Twelve patients were excluded because of a follow-up period of less than 6 months (lost to follow-up or died due to their tumor within 6 months after resection). Hence, complete data of 162 patients were available for analysis. The median follow-up time was 55.6 months (mean 52.9, range 7–103 months). Eighty-seven patients (53.7%) were male and the median age of all patients was 52.9 years (mean 50.0, range 10–86 years). Further characteristics of the patient cohort are summarized in Table 1; anatomic distribution of tumor sites and entities are demonstrated in Figs. 1 and 2. One hundred thirty-seven biopsies (84.6%) were performed as CNB and 25 (15.4%) as IB. Seventy tumors (43.2%) were located in the bone and 92 (56.8%) in soft tissue.

**Table 1:** Characteristics of the patient cohort (^a^13 Ewing sarcomas were excluded of grading classification)

		Number	Percentage
Total patients		162	100
Median age (years)		55.6	
Type of biopsy	CNB	137	84.6
	IB	25	15.4
Tissue type	STS	92	56.8
	BS	70	43.2
Grading^a^	G1	24	14.8
	G2/3	125	77.2
Number of LR		22	13.6
Median time to LR (months)		22.4	
Perioperative radio-/chemotherapy		118	72.8

Radio-/chemotherapy (in some cases combined with local hyperthermia) was performed in 118 cases (72.8%). Twenty-two patients (13.6%) developed local recurrence in a median of 22.4 months (mean 28.7, range 4–48) after tumor resection.

**Fig. 3:**
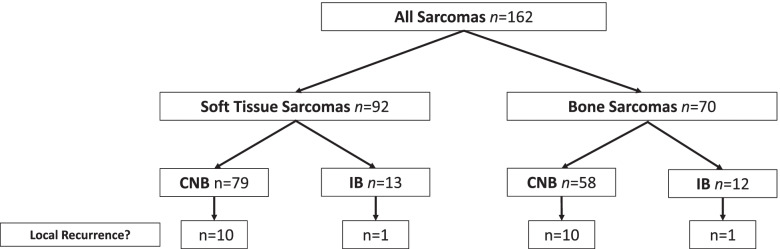
Characteristics of the tumors regarding the type of tissue and biopsy. CNB core-needle biopsy, IB incisional biopsy

The cohort was divided into two groups with respect to the type of biopsy: CNB and IB (Fig. 3). Both groups were comparable regarding the major clinical factors as kind of tissue (*p* = 0.254), grading (*p* = 0.069), and perioperative therapy (*p* = 0.681). There were 20 LR in 137 cases (14.6%) in patients with CNB and 2 LR in 25 cases (8%) of IB (*p* = 0.376).

### Bone sarcomas

**Fig. 4:**
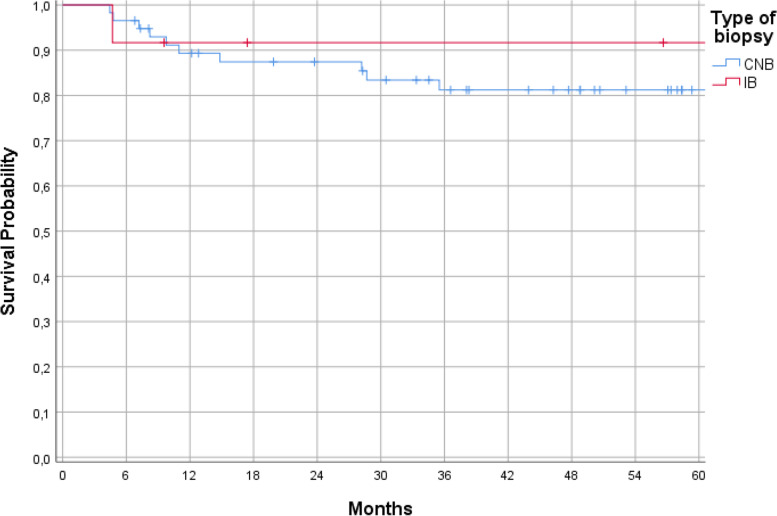
Local recurrence-free survival depending on the type of biopsy in bone sarcomas (*p* = 0.476).

Seventy patients with bone sarcomas were included in this study. Fifty-eight (82.9%) biopsies were performed as CNB and 12 (17.1%) as IB. There were 11 (15.7%) cases with a LR. Ten (17.2%) of these occurred after CNB and one (8.3%) after IB (*p* = 0.675). The median LRFS of all patients with bone sarcomas was 54.9 months (range 4–104 months). The median LRFS in CNB was 49.5 months and in IB 69.1 months (*p* = 0.476; Fig. 4). Seven of 11 LR were chondrosarcomas (23.3% of all chondrosarcomas), 3 osteosarcomas (11.5%), and 1 Ewing sarcoma (7.7%) (*p* = 0.440).

Grading and perioperative multimodal therapy are factors that potentially could have influenced the occurrence of LR independently of the type of biopsy. They were evaluated in a univariate analysis and showed no significant influence on LR rate in this highly selected group of patients (grading *p* = 0.224; multimodal therapy *p* = 0.448).

If grading and multimodal therapy were used as separating factors, the type of biopsy also showed no influence on LR risk (3 groups of grading *p* = 0.440; two groups of multimodal therapy *p* = 0.395). Multivariate analysis of the data returned the same result (grading *p* = 0.442, 95% confidence interval −0.219–0.097; multimodal therapy *p* = 0.859, 95% confidence interval −0.220–0.263). The type of biopsy also had no influence within the subgroups of chondrosarcoma (*p* = 0.468), osteosarcoma (*p* = 0.592), and Ewing sarcoma (*p* = 0.923).

### Soft tissue sarcomas

Ninety-two patients with STS underwent biopsy and sarcoma resection. The biopsy was performed as CNB in 79 patients (85.9%) and 13 (14.1%) underwent IB. LR occurred in 11 (12%) cases. In 79 CNBs, 10 (12.7%) cases with LR were seen; in 13 IBs, one LR (7.7%) was evident (*p* = 0.517). Six of 11 LR were non otherwise specified (NOS) sarcomas, 2 malignant peripheral nerve sheath tumor (MPNST), and each one fibrosarcoma, rhabdomyosarcoma, and leiomyosarcoma.

**Fig. 5:**
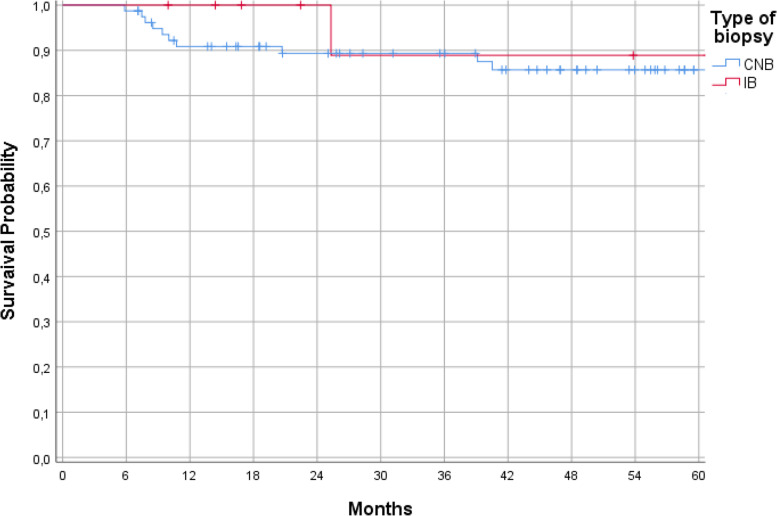
Local recurrence-free survival depending on the type of biopsy in soft tissue sarcomas (*p* = 0.611)

The comparison of LR rate between low-grade and high-grade tumors showed a trend towards a higher rate in high-grade sarcomas (13.8% vs 0%); however, this trend did not reach significance (*p* = 0.195). Multimodal therapy also did not influence the risk of LR (*p* = 0.472). The median LRFS in CNB was 48.5 months and in IB 60.9 months (*p* = 0.611; Fig. 5).

Also in STS, there was no significant influence of tumor grading (*p* = 0.517) and multimodal treatment (*p* = 0.505) on the rate of LR in both types of biopsies using univariate analysis. Multivariate analysis for grading (*p* = 0.165, 95% confidence interval −0.173–0.030) and perioperative therapy (*p* = 0.869, 95% confidence interval −0.213–0.180) showed the same.

## Discussion

A multidisciplinary approach in the therapy of STS and BS is well-established [16, 17]. The influencing risk factors for survival of the patients as well as clinical outcomes have been the subject of some studies. However, the number of publications investigating the role of biopsy for the risk of local recurrence in sarcoma treatment is limited. The basis of this discussion is findings in the histopathological evaluation of the biopsy tracts with contaminating tumor cells. Some published data clearly show a significant difference between a high tumor cell load in an IB tract and a hardly existent tumor cells in CNB tracts [6, 18]. Therefore, there is a change in opinion regarding the excision of biopsy tracts in CNB and an increasing number of authors are not advocating such a strategy, including our institution [4, 7]. However, those studies included also cases with positive resection margins.

We were able to investigate a homogenous cohort of sarcoma patients, which underwent diagnostic and treatment procedures in a dedicated sarcoma center under the care of only two surgeons. Due to exclusion of patients with “whoops surgery,” positive margins, and non-standard treatment, we resulted in a good comparability of subgroups.

We were unable to identify any significant differences between CNB and IB regarding LR risk within the whole cohort and within individual subgroups. Our results do not show a significant difference in the LR rate between CNB and IB; however, we observed a trend towards a disadvantage of CNB with LR in 14.6% of cases, compared to 8% in IB cases. This finding represents a contradiction to the published literature: Barrientos-Ruiz et al. [6] showed a significantly higher degree of biopsy tract seeding with tumor cells in the case of IB. In their study, the biopsy tract was resected in every case, also in CNB. However, at the end, an IB was associated with an increased risk of LR and the study by Oliveira et al. found the same results [8]. One might conclude that non-resection of the biopsy tract in cases with CNBs in our study cohort causes the non-significant, but in trend higher rates of LR. The literature stands opposed to this perception. Berger-Richardson et al. [9] pooled 3 studies with a total of 63 patients in their review. In one of those studies, a contamination of excised biopsy tracts after CNB was seen in 13%. In another described cohort with 30 patients without excision of the biopsy tract after CNB, there were no cases of LR. Not excising the biopsy tract after CNB carried a risk of LR of 8% in the study by Binitie et al. [4]. The authors concluded, the same number in other studies including patients with resected biopsy tracts prove no influence on LR rate.

The risk of LR depends on many factors, such as entity, grading, and multimodal therapy. The value of multimodal therapy and its influence on the local recurrence rate is well known [16, 19]. However, there are tumor entities without further therapy options, except surgery (e.g., chondrosarcoma, superficial low-grade soft tissue sarcomas). In both groups and with or without multimodal treatment, we did not see any significant influence of the biopsy type on the LR rate or on LRFS.

In addition, the influence of grading on the LR rate is also well established. In cases with low-grade sarcoma, the risk of LR is significantly lower [20, 21]. Due to the low numbers of patients in this particular subgroup, we were unable to duplicate these findings in our highly selected study group. However, within both subgroups of low- and high-grade tumors, almost identical results for CNB and IB were seen.

The biological characteristics and behavior of bone and soft tissue sarcomas are different [22, 23]. However, we did not observe any differences between STS and BS regarding the type of biopsy. The evaluation of differences in entity subgroups of STS was not feasible because of the high number of entities and the consecutively very small numbers of patients in each subgroup. The subgroup analysis in BS showed a high rate of LR in chondrosarcomas (23.3%); however, this difference did not reach statistical significance in relation to other entities. A possible explanation for the observed difference could be the location of these tumors. Chondrosarcomas often arise in the pelvis with known worse results as compared to LR in extremity tumors. Atypical cartilaginous tumors (chondrosarcomas G1 of the extremities) did not undergo biopsy and were diagnosed by correlation of radiological and histological investigation results. Those cases are hence not included in this study.

Until today, published studies have shown that CNB and IB provide similar sensitivity regarding the diagnosis of sarcomas [3] and that IB has a higher risk for complications [24] and a potentially higher risk of spreading tumor cells [6]. The excision of IB tracts with tumor resection causes larger soft tissue defects and a higher risk of complications [2, 25].

The most important factor for lowering the risk of LR in incisional biopsy seems to be the excision of the biopsy tract. Our analysis of IB and CNB demonstrate an equivalence of both biopsy methods regarding the risk of LR and of LRFS, even if the biopsy tract was not excised in CNB.

## Conclusions

We were unable to demonstrate different rates of LR or LRFS in patients with excised IB tracts and patients with non-excised CNB tracts in bone and soft tissue sarcomas of the extremities. Additional factors such as multimodal treatment (including radiotherapy) and grading did not influence this result. Based on our results, the excision of the biopsy tract in cases of CNB does not appear to be necessary.

## Data Availability

The datasets used and/or analyzed during the current study are available from the corresponding author on reasonable request.

## Abbreviations

CNB: Core-needle biopsy
IB: Incisional biopsy
LR: Local recurrence
LRFS: Local recurrence-free survival
OS: Overall survival
CT: Computed tomography
MRI: Magnetic resonance imaging
PET/CT: Positron-emitting tomography-CT
STS: Soft tissue sarcoma
BS: Bone sarcoma
